# Electron Spin Resonance (ESR) Dating of Calcareous Fault Gouge of the Ushikubi Fault, Central Japan

**DOI:** 10.1007/s00723-013-0471-9

**Published:** 2013-07-10

**Authors:** Emilia Bi Fantong, Akira Takeuchi, Ryosuke Doke

**Affiliations:** 1 Graduate School of Science and Engineering, University of Toyama, 3190 Gofuku, Toyama, 930-8555 Japan; 2 Hot Spring Research Institute of Kanagawa Prefecture, 568 Iriuda, Odawara, Kanagawa 250-0031 Japan

## Abstract

The ages of fault events of active faults have been estimated using electron spin resonance (ESR) signals of siliceous gouges. This technique of ESR method is limited by obtaining only ages that are greater than tens of millennia. So this study focuses on developing a new technique of using calcareous gouges to gain an insight into the ages of latest seismogenic event within the Holocene. For the first time, signal B of the ESR method has been used to estimate the age of the Ushikubi fault from calcareous gouge. This technique proved reliable because the mean age (1.9 ka) obtained agrees with previous works on indirect age determination of latest fault events by utilizing radiocarbon dating in the study area. However, the result from the ESR technique showed an increase relative to the age of 1 ka that was obtained by the radiocarbon dating method. This disparity may be due to a high dose rate value of 50 Gy/h of artificial irradiation that was used to determine the equivalent dose (ED). Moreover, isochronal experiment revealed that the gouge did not comprise pure carbonates but consisted of a mixture of calcite and quartz grains. A younger age value would have been obtained if a lower artificial irradiation dose rate and a relatively pure carbonate fault gouge were used in the ED determination.

## Introduction

Active tectonics is associated with uplifts, earthquakes, volcanic eruptions, landslides and faulting, which have been reported having a direct impact on the environment and population [1, 2]. One of the countries in the World that is most vulnerable to the aforementioned components of active tectonics is Japan [3, 4]. The environmental and human impacts from the incidence of active tectonics can among others be exemplified by the March 11, 2011 earthquake and tsunami along the northeastern coast of Japan, and the 2007 Niigata Chuetsu-Oki earthquakes. These underscore the importance of monitoring of active tectonics. The obtained data shall therefore contribute in the process of identifying strategies to reduce earthquakes/fault-related disasters by decision makers. In recognition of this importance, earth scientists have not only intensified but also refined on both spatial and temporal dimensions the characterization of faults and earthquake prone zones in Japan. As far as temporal characterization is concerned, quartz in fault gouges has been used to estimate the age of latest fault movements using the electron spin resonance (ESR) method [5–8]. The established ESR method reported by Ikeya et al. [5] showed the possibility of dating young fault based on the presence of defect centers that are formed in quartz grains within fault gouges. Among these defect centers, a paramagnetic center called $E_{1}^{\prime }$ (oxygen vacancy with one electron) center has been used extensively to characterize and date faults. However, ESR results gotten through this signal are unlikely because of the following reasons: this signal is usually associated with a counterfeit signal [7] and is also associated with another signal called the *R* signal which has a short spin lattice relaxation time [8]. Moreover, ages <10 ka have never been gotten in defect centers present in quartz grains in fault gouges. Irrespective of all attempts to date young faults with the ESR method using defect centers in quartz, the main limitation reported by Noller et al. [9] and Ikeya et al. [10] is that this method lacks the resolving power of rocks younger than 2 Ma.

Because of the importance of understanding active faults and estimating the age of faults to improve upon the mitigation management and hazard assessment [11], various investigations by various methods have been employed to elucidate the recent history and activity of the Ushikubi fault in central Japan. Some of these investigations include: the use of iron speciation [12], the use of geomorphology and geology [13, 14], and the use of the radiocarbon dating method [15–17]. Although the age of the latest event of the Ushikubi fault has been estimated indirectly using the radiocarbon dating method, the ESR method has not yet been employed.

Accordingly, this study employs a novel approach of using calcareous gouge as an active fault dating material to supplement the existing methods with the main objective to identify a useful ESR signal for dating of calcareous fault gouge and also develop a method to determine the age of the latest seismogenic event of the Ushikubi Fault in central Japan.

## Location, Physiography, and Geology of the Study Area

**Fig. 1:**
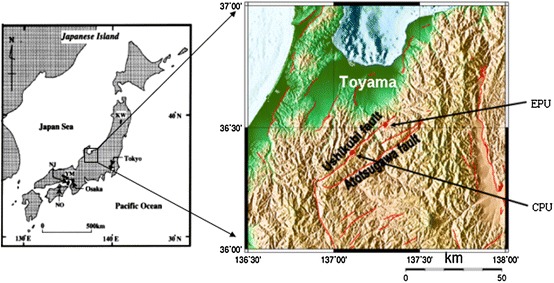
Index map showing the location of the Ushikubi fault and sample locations in Japan (modified from [30]) *EPU* the eastern part of the Ushikubi fault, *CPU* the central part of the Ushikubi fault

**Fig. 2:**
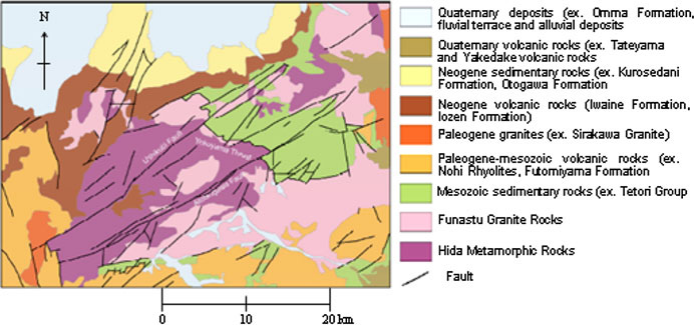
Geological map around the northern margin of the Hida highland (edited from [28, 29])

The study area (Ushikubi fault), which is 52 km long NE–SW right lateral trending fault, is part of the Atotsugawa fault system that is in the northern margin of the Hida Highland, central Japan. It lies within latitudes 36°15′N and 36°30′N and longitudes 137°E and 137°30′E (Fig. 1). This area shows a rugged relief, with incised valleys, which are drained by the Jinzu, Joganji and Shou rivers [13]. The northern margin of the Hida highlands belongs to the Hida geologic belt. The basement consists of Paleozoic Hida metamorphic rocks (felsic gneiss, hornblende gneiss, meta-mafic rocks, crystalline limestone and calcareous gneiss that contain biotite). These basements are intruded by Jurassic Funatsu granites which are overlain by Cretaceous sedimentary rocks of the Tetori group (sandstones, mudstones, conglomerates) [13] (Fig. 2).

## Method of Study

**Fig. 3:**
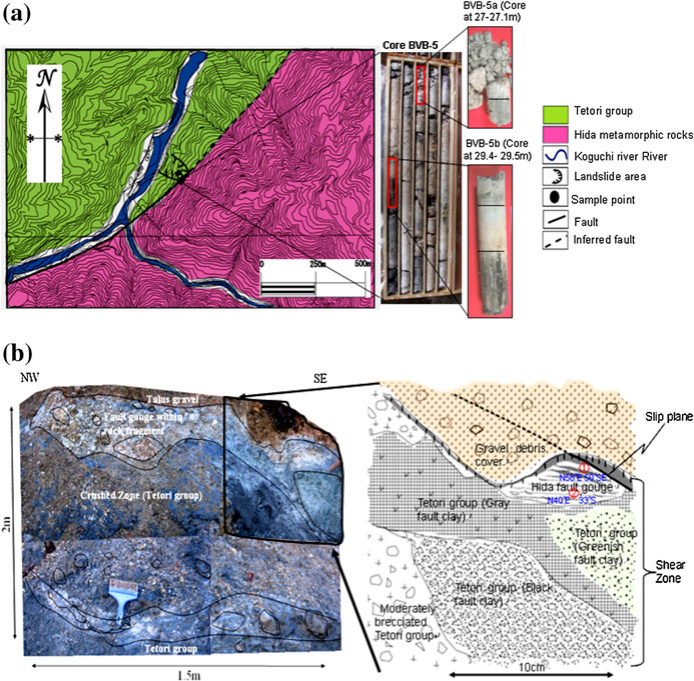
**a** Geologic map of the eastern part of the Ushikubi fault indicating sample site (*two black dots*). *Photographs on the right* represent the core samples that were used in this study. The *red rectangles* show areas where samples were taken at different depth (BVB-5a at 27–27.1 m and BVB-5b at 29.4–29.5 m). *Black lines* across the core samples demarcate different formations. The samples of interest were taken from the uppermost layer of the different core samples. **b** Landslide scar along the eastern part of the Ushikubi fault (*left*) and a sketch of part of the outcrop (*right*) showing sample collection site at ① and ②. The sketch corresponds to the area in the form of a trapezium with *thick black lines*. Samples were collected within the light-colored gouge layers at a depth of about 20 cm. This *shear zone* represents relics of the Tetori group and the Hida metamorphic rocks that were crushed to form the gouge (color figure online)

**Fig. 4:**
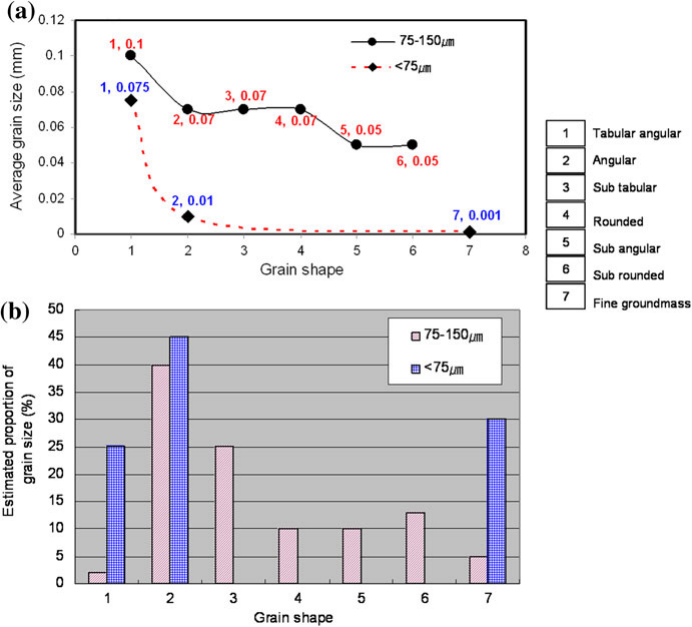
Relationship between grain size and grain shape in the calcareous fault gouge. Graph **a** shows a decrease in grain size as a function of grain shape in both the 75–150 and <75 μm. Larger grains are mostly tabular to angular in shape and fine grains constitute the ground mass. Graph **b** shows angular grains dominating in both fractions. Grain shapes could not be described when the size is <75 μm

**Fig. 5:**
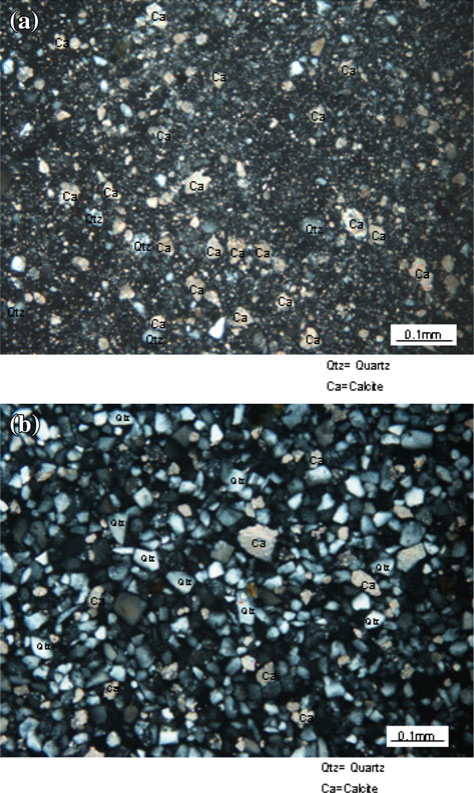
**a** Micrograph of <75 μm calcareous fault gouge. The proportion of calcite (*Ca*) in this size fraction is greater than that of quartz (*Qtz*). **b** Micrograph of 75 to <150 μm calcareous fault gouge. The proportion of quartz (*Qtz*) in this size fraction is greater than that of calcite (*Ca*). Thin sections were prepared using microslide glass with thickness of 1.3 mm. Observation and photographs were done using an OPTIPHOT 2-POL plane polarized microscope (Camera head; DS-5 m and control unit; DS-L1)

**Fig. 6:**
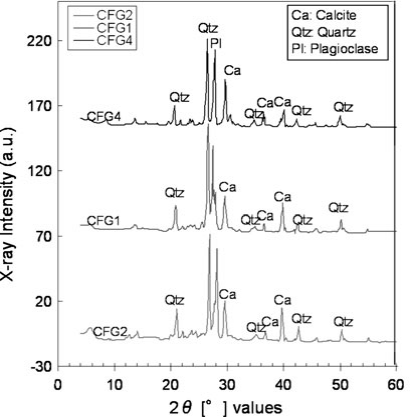
X-ray diffraction pattern obtained from calcareous fault gouge samples from the central part of the Ushikubi fault (CFG1, CFG2 and CFG4). *Ca* calcite, *Qtz* quartz, *Pl* plagioclase, voltage was 40 kV and current was 20 mA, chart speed was 40 mm/min, angle ranged between 60 degree and 4 degree

The method of the study was conducted in two phases: field work and sampling, and laboratory analyses. Field work and sampling were conducted in July 2008 when four calcareous fault gouge samples (CFG1, CFG2, CFG3, and CFG4) were collected from the central part of the Ushikubi fault at shallow depths of about 20 cm, and three were collected from the eastern part (East.CFG1, East.CFG2, East.CFG3). Two core samples BVB-5a and BVB-5b were collected at a depth of 27–27.1 and 29.4–29.5 m, respectively, from the eastern part of the Ushikubi fault. The sites from where the various samples were collected are shown in Figs. 1 and 3a. These samples were collected along the eastern part of the Ushikubi fault in an area that was exposed by a landslide along a slip plane (strike N58°E and dip 50°SE) (Fig. 3b). The fresh landslide scar exposed weathered and crushed remains of the Tetori group, gravel layer and relics of the Hida metamorphic rocks. The Tetori group in this outcrop is represented by sandstones and mudstones which have been crushed to form breccias, cataclasites and fault gouges. The gravel layer in this outcrop consists of angular to sub-angular fragments of the Hida metamorphic rocks which vary in size from 1 to 60 cm. Fragments of the Hida metamorphic rocks and the Tetori group, which ranged in size from 3 to 57 cm, were also observed in the shear zone. The fault gouge within this zone exhibits a hue of colors such as grayish black, bluish gray, greenish, pinkish and whitish. Figure 3b shows the sketch diagram of part of the outcrop exposed by landslide. Fresh samples were carefully collected from the light-colored portion of the fault gouge with the intention of obtaining calcite-rich gouges. After sampling, the samples were then washed with distilled water and then allowed to dry in a dark room. The dried samples were then sieved to obtain fraction size between <75 and 75–150 μm. With the aid of an electron microscope, it was observed that the sieved grains varied in shape such as tabular, angular, sub-angular to angular and sub-rounded to rounded. The estimated proportion of the different grain shapes was as shown in Fig. 4, which shows that the angular grains were dominating. Micrographs of the fractions <75 and 75–150 μm were also taken as shown in Fig. 5a, b to verify and confirm the presence of calcite in the fault gouge. X-ray diffraction patterns also indicate the presence of calcite in the fault gouge (Fig. 6). The obtained calcareous gouge was then analyzed in the laboratory with the ESR method.

### Laboratory Analyses

The samples were subjected to ESR analyses at the University of Toyama, Japan.

Prior to ESR analysis, the gouge samples were washed with distilled water and then dried in a dark environment to avoid possible effects of sunlight on the ESR signal intensity [10]. After that, the dried portions were then sieved to obtain grain size that ranged from 75 to <150 μm because this study focused on investigating gouge-sized samples. More over, calcite with a hardness of 2.6 is more susceptible to crushing than quartz with a hardness of 7. Colored and sizable grains were also removed by hand picking with the aid of a microscope. The samples were then tested with dilute HCl to verify and confirm the presence of carbonate in the gouge. An ESR spectrometer operating at an X-band frequency was the typical spectrometer that was used in this study. Aliquots (100–250 mg) of each size fraction was placed in 4 mm quartz tube and ESR analysis were conducted using a JEOL JES-REIX, X-band spectrometer operating at a modulation frequency of 100 kHz and a microwave power of 1 mW. Other instrumental conditions that were considered during the experiment include an optimal central field range of 334–335 mT, sweep width of 4–7.5 mT over 10 s, field modulation of 1.25 × 0.01 mT, time constant of 0.3–1 s, and receiver gain of 7.9 × 100. The purpose for using these conditions was to enable comparison with other spectra of the same species. Moreover, with those conditions, the reproducibility of the signal intensities for the different samples yielded better results after conducting a series of control experiments on signals of the same species. A sweep width of 7.5 mT was chosen because at this condition, it was possible to record both the signal of interest, and the third and fourth lines of manganese, which were used for the calibration of the signal intensity. With a sweep width <7.5 mT, it was possible to measure only the signal of interest without measuring the third and fourth manganese lines. However, in situations where the signal of interest needed close observation, a sweep width of 4 mT was used. The intensity of each signal in each sample was then taken as the peak-to-peak height. The ESR intensities and *g* factors of the signals were calibrated using the average intensity of the third and the fourth Mn^2+^ lines as shown in Eq. (1)

1 $$ I_{\text{f}} = 100 \times I_{\text{o}} /{\text{Mn}}^{\text{Av}} , $$

where *I*_f_ is the final signal intensity, *I*_o_ the initial signal intensity and Mn^Av^  is the average of third and fourth line of Mn^2+^ intensity.

The thermal experiment consisting of isochronal annealing of 15 min from 50 to 450 °C was performed with an Isuzu Muffle oven. The heated samples were then allowed to cool down in desiccators to avoid the absorption of moisture.

Artificial irradiation of the calcareous gouge samples was performed using ^60^Co γ-ray source at a dose rate of 50 Gy/h at the Osaka prefecture University, Japan. The samples were then analyzed with the ESR spectrometer under the above-mentioned conditions at room temperature. The obtained results were then used for the determination of the equivalent dose (ED) (Gy). Annual dose rates (*D*) (Gy/ka) were adopted from the data of [6] from Atotsugawa fault on the assumption that annual dose rate in ordinary environment is relatively constant at ground level [10]. ESR ages were then calculated using the relationship,

2 $$ T_{\text{ESR}} = {\text{ ED}}/D, $$

where *T*_ESR_ is the ESR age, ED the equivalent dose, and *D* is the annual dose rate.

Thin sections used in this study were prepared using microslide glass with a thickness of 1.3 mm. Samples were observed and photographs were taken using an OPTIPHOT 2-POL plane polarized microscope (camera head; DS-5 m and control unit; DS-L1).

X-ray diffraction patterns that are used in this study were measured with a RIGAKU GEIGERFLEX diffractometer with voltage and current of 40 kV and 20 mA, respectively.

## Results

### ESR Spectra of Naturally Irradiated Calcareous Fault Gouge

**Fig. 7:**
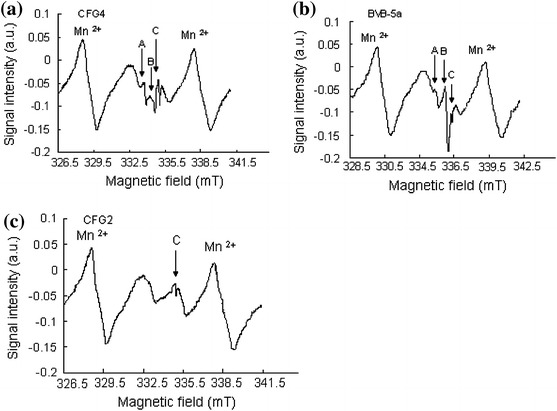
**a** ESR spectrum of sample CFG4 (natural) measured at room temperature. Signals A, B and C are ascribed to ${\text{SO}}_{2}^{\cdot{ - }} ,\;{\text{SO}}_{3}^{\cdot{ - }} ,\;{\text{CO}}_{2}^{\cdot{ - }}$, respectively. **b** ESR spectrum of sample BVB-5a (natural) measured at room temperature. **c** ESR spectrum of sample CFG2 (natural) measured at room temperature showing only one peak (signal C). Measurement conditions were: microwave frequency 9.43 GHz, sweep width 7.5 mT, microwave power 1 mW, modulation frequency 100 kHz, time constant 0.3 s, sweep time 8 min, field modulation 1.25 × 0.01, receiver gain 7.9 × 100

Figure 7a–c represents the ESR spectra obtained from natural calcareous fault gouge samples measured at room temperature. Three distinct peaks (signals) were identified in both the surface and the core samples. These identified signals were tentatively ascribed to A (*g* = 2.0060), B (*g* = 2.0033) and C (*g* = 2.0007) in the surface samples and A (*g* = 2.0066), B (*g* = 2.0022) and C (*g* = 2.0003) in the core samples. Sample CFG2 showed only one well distinct peak at *g* = 2.0007 when measured at room temperature (Fig. 7c). Although the *g* factors (*g* = 2.0007) of the signal (e.g., signal C) in the surface samples is different from those of the core samples (*g* = 2.0003), they fall within the range of *g* values for the C signal in carbonates (*g* 2.0003–2.0009) [18, 19]. It is worth noting that to recognize a signal base on its *g* value only is not sufficient to label a paramagnetic center [20].

**Fig. 8:**
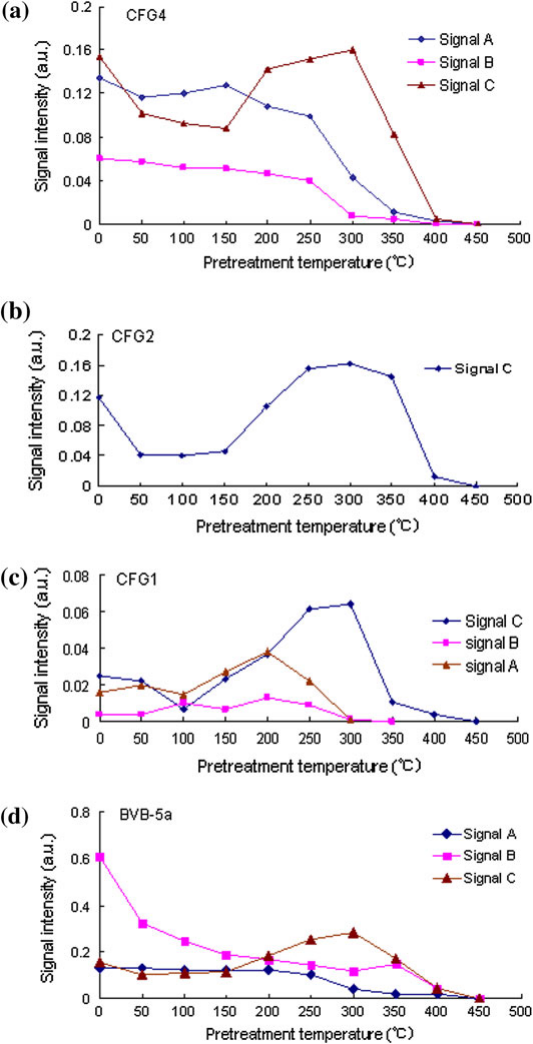
**a** Isochronal curves showing variation in signal intensity with temperature in sample CFG4. The ESR intensities of signals A and B decrease with increasing temperature and anneal out completely at 450 °C. Signal C showed a unique behavior in its decay pattern in which the first decrease in signal intensity was observed as temperature was increased to 150 °C and the second from 300 °C and finally anneals out at 450 °C. This unique behavior in the decay pattern of signal C in the different samples is attributed to the presence of $E_{1}^{\prime }$ defect center in quartz whose intensity increases with increase in temperature. The heating duration was 15 min with an internal temperature from 50 to 450 °C in all the annealing experiments. The annealing experiment was done in an Isuzu muffle oven. **b** Isochronal curves showing variation in signal intensity with temperature in sample CFG2. The ESR intensity of signal C equally showed a unique behavior. In this case, the first decrease was observed at a temperature of about 100 °C and the second from 300 °C. Complete annealing was observed at 450 °C. **c** Isochronal curves showing variation in signal intensity with temperature in sample CFG1. The ESR signal intensity of signal A decreased with increasing temperature up to 100 °C. Its intensity increased a little bit as temperature was raised to about 200 °C. This signal annealed out completely at about 300 °C. Signal B, decreased with increasing temperature and annealed out completely at 300 °C, while signal C equally decayed in two stages. **d** Isochronal curves showing variation in signal intensity with temperature in sample BVB-5a. A general decrease in the intensities of signals A and B was equally observed in this sample while that of signal C decreased in two stages as the temperature was increased. Complete annealing of all the three signals was observed at 450 °C

**Fig. 9:**
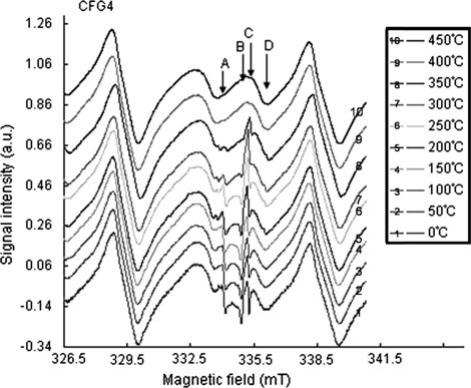
Isochronal ESR spectra of sample CFG4 showing additional peak as indicated by *D*. This signal appeared at about 200 °C and its intensity decreased gradually and systematically with further increase in temperature

Because of the difficulties in distinguishing and assigning *g* values to various paramagnetic defect centers in the samples, isochronal annealing experiments were performed on some of the samples (CFG4, CFG2, CFG1 and BVB-5a). This experiment revealed that the patterns of signal intensity with increasing temperature for the signal at *g* = 2.0007 were unique in behavior in all the samples including those showing only one peak (Fig. 8a–d). From the isochronal curves, a decrease in signal intensity occurred in two stages as shown in Fig. 8. A general decrease in the signal intensity was first observed from 0 to 150 °C, and as the temperature was gradually increased from 150 to 300 °C, the intensity of the peak increased gradually. The second decrease was observed from 300 to 450 °C. The number of peaks (signals) observed in samples heated for up to 150 °C was the same as with those of the natural (not heated) measured under the same conditions. As the temperature was raised from 200 to 350 °C, the number of peaks became more than those of their respective natural room temperature counterpart in some of the samples as indicated by signal D (*g* = 1.9968) in Fig. 9. However, this signal is unstable and its intensity decreased gradually and systematically from 350 to 450 °C. The decay pattern of the signals at *g* = 2.0007 and *g* = 2.0003 in the surface samples and core samples (BVB-5a), respectively, suggests that the samples are made of more than one defect center. This behavioral pattern revealed that the signal at *g* = 2.0007 and *g* = 2.0003 is most likely a mixture of both the $E_{1}^{\prime }$ paramagnetic centers in quartz and C (${\text{CO}}_{2}^{\cdot{ - }}$) signal in carbonates.

### ESR Spectra of Artificially Irradiated Calcareous Gouge

**Fig. 10:**
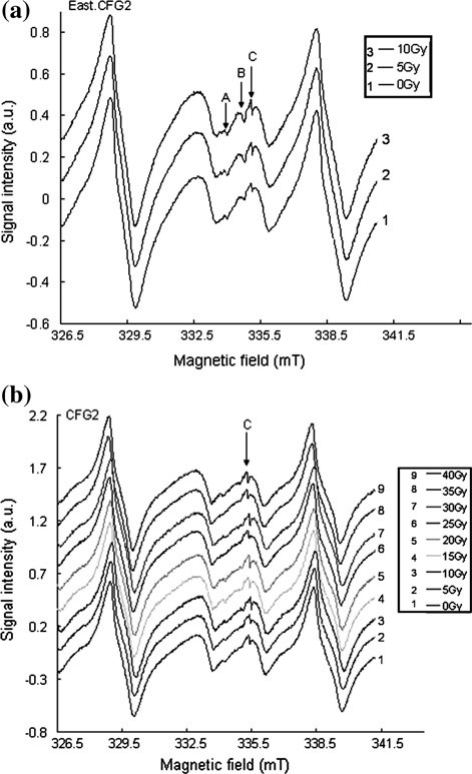
**a** ESR spectra of sample East.CFG2 showing increase in the intensities of signals A, B and C at a dose of 0, 5 and 10 Gy. **b** ESR spectra of sample CFG2 showing an anarchical variation in the intensity of signal C with increasing dose rate. From 0 to 10 Gy, there is growth in signals A, B and C, and between 15 and 40 Gy, a variation in the intensities of the different signals is observed

**Fig. 11:**
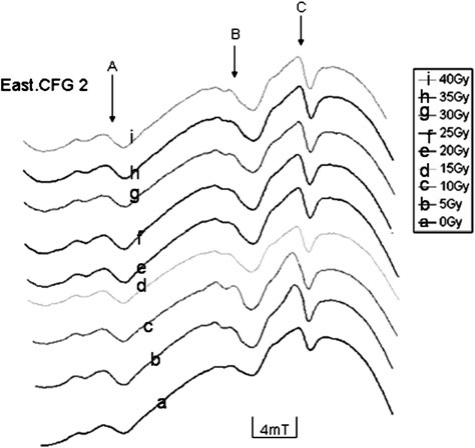
Summary of the saturation behavior of the ESR spectra (e.g., East.CFG2) with increasing dose measured at 4 mT over a sweep time of 16 min. The intensities of signals B and C are enhanced at low levels of added artificial irradiation dose (0–10 Gy) while that of A is not greatly enhanced. Upon addition of artificial irradiation dose (15–40 Gy), no growth in the intensities of the different signals is observed. When signal intensity does not increase upon addition of artificial irradiation dose, it means signal are saturated

The ESR spectra of the artificially irradiated samples (East.CFG1, East.CFG2, CFG1 and CFG4) measured at room temperature are equally characterized by the presence of three distinct peaks (signal A, B and C), while samples CFG2 showed only one peak (signal C) (Fig. 10a, b). Neither pre- nor pro-thermal treatment was done on the artificially irradiated samples. At lower levels of artificial irradiation, the intensities of the different signals were enhanced and there was no growth with increasing irradiation dose implying signal saturation. As dose rate was increased from 0 to 10 Gy with an interval of 5 Gy, the signal intensity increased in all the samples (Fig. 10a). A decrease in signal intensity was observed in some of the peaks (e.g., signal C) in sample CFG2 as the dose was added up to 15 Gy (e.g., Fig. 10b). When the irradiation dose was increased from 20 to 40 Gy, the intensity of the signal varied in an anarchical manner (Fig. 10b). Although an increase in signal intensity was observed at a lower dose range, some signals got saturated as the dose was increased. The overall observation in the saturation behavior of some of the signals with increasing dose is summarized in Fig. 11, which showed no growth in the intensity of the different signals with increase in the irradiation dose.

## Discussion

### Signals in ESR Spectra

**Fig. 12:**
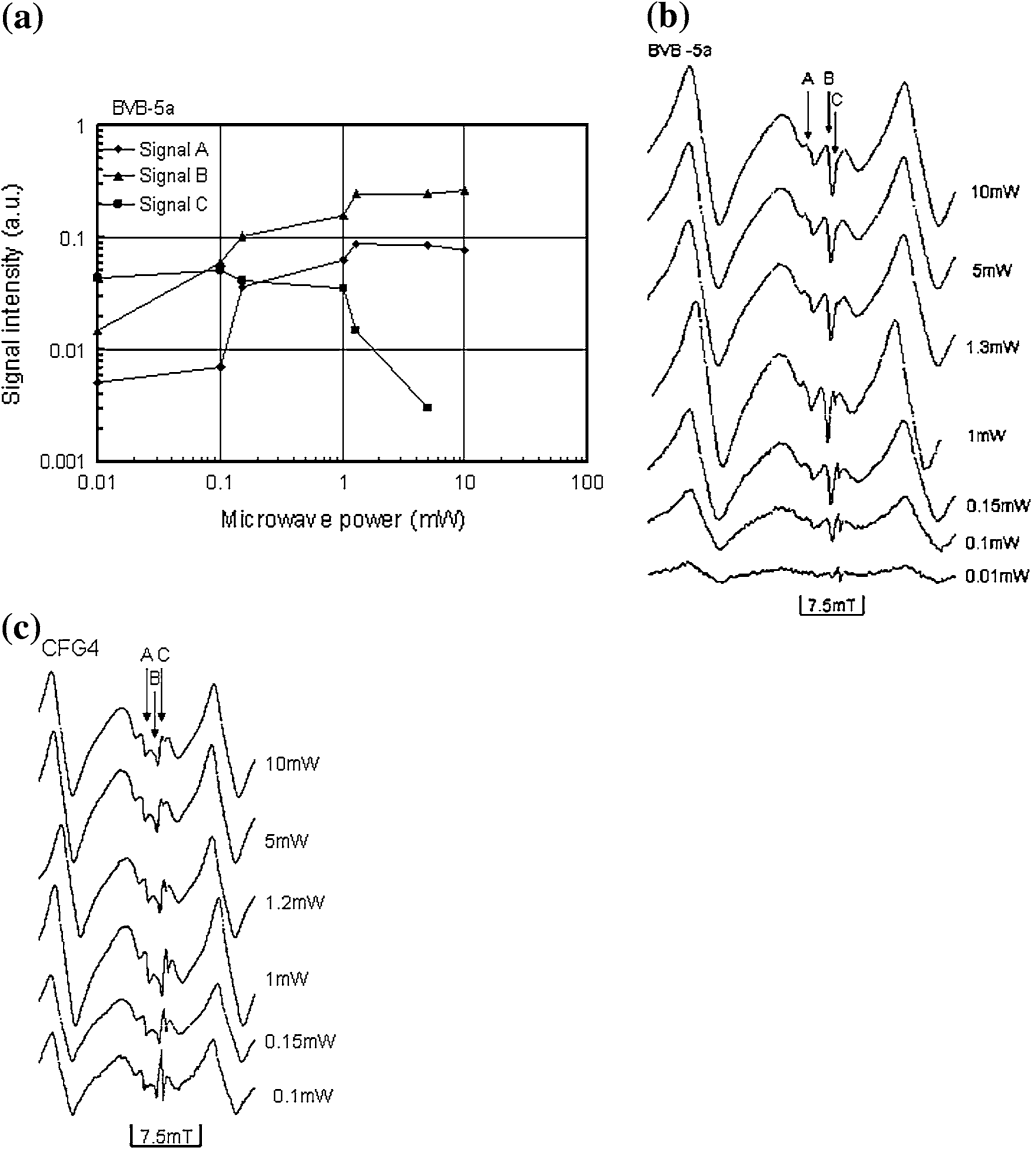
**a** Microwave power dependence of signals A, B and C in sample BVB-5a. Signal C saturates at a moderate to high microwave power, while signals A and B do not saturate even at high microwave power. The $E_{1}^{\prime }$ center in quartz saturates at a relatively lower microwave power; however, at a microwave power of 1 mW, this signal is still visible due to the presence of the signal C in carbonates. **b** ESR spectra of signals A, B and C in BVB-5a measured at different microwave power. **c** ESR spectra of signals A, B and C in CFG4 measured at different microwave power. The saturation pattern of the different signals are the same in the different samples investigated, e.g., in CFG4 signal C is still detectable at 1 mW, while in BVB-5a, this same signal is not visible implying that the proportion of carbonate in CFG4 is more than in BVB-5a. However, the degree of saturation varies among different signals in different samples

The A, B and C signals are attributed to the ${\text{SO}}_{2}^{\cdot{ - }} ,\;{\text{SO}}_{3}^{\cdot{ - }} ,\;{\text{and}}\;{\text{CO}}_{2}^{\cdot{ - }}$, respectively [10, 21]. The signal C corresponding to the ${\text{CO}}_{2}^{\cdot{ - }}$ is the most frequently used signals in carbonate dating [10, 21]. From the isochronal curves, it can be seen that this signal anneals out completely at about 100–150 °C (Fig. 8a–d). These results are consistent with the observations, as reported by others [10, 21–23], that in carbonates, the C signal (${\text{CO}}_{2}^{\cdot{ - }}$) anneals out completely at about 200–250 °C. The slight variation in the temperatures could be as a result of an additional defect center ($E_{1}^{\prime }$ center) since the samples contained a mixture of quartz and calcite as shown in the micrograph of Fig. 5a, b. One possible explanation for the variation in the temperature could also be that the samples investigated are different from those previously investigated by other researchers (carbonates in fossils, speleothem, fault gouge, etc.). The intensity of the $E_{1}^{\prime }$ defect center in quartz increases above 150 °C as reported by Toyoda and Schwarcz [7] and Ikeya et al. [10]. From the isochronal curves (Fig. 8a–d), it can be observed that the intensity of the peak at *g* = 2.0007 and *g* = 2.0003 (attributed to the C signal) increased with increasing temperature up to about 300 °C and annealed out completely at about 450 °C. These results are consistent with the observation of Toyoda and Schwarcz [7] and Ikeya et al. [10]. Moreover, at a low to moderate microwave power, its dependency showed a saturation behavior of this peak as shown in Fig. 12a, c, such phenomenon confirms the presence of the $E_{1}^{\prime }$ defect center. The $E_{1}^{\prime }$ defect center in quartz saturates easily at low microwave power as reported by Hataya et al. [8]. However, at a relatively higher microwave power of 1 mW, the intensity of this peak is still visible indicating the presence of another signal which is most probably the C signal in carbonates (Fig. 12c). On the other hand, signals with *g* = 2.0060 attributed to the ${\text{SO}}_{2}^{\cdot{ - }}$ corresponds to the same signal at *g* = 2.0056 [10]. This signal is not greatly enhanced upon artificial irradiation (Fig. 10a). The intensity of this signal increased with increasing temperature at the expense of signal B (Fig. 8c). The microwave power dependence of this signal shows that this signal does not saturate even at a moderate to high microwave power (Fig. 12a–c). This observation is supported by the findings of Ikeya et al. [10]. The signals with *g* = 2.0033 attributed to the ${\text{SO}}_{3}^{\cdot{ - }}$ correspond to the same radical species at *g* = 2.0036 [21, 24], at *g* = 2.0032 [25], and at *g* = 2.0035 [10, 26]. This signal is enhanced by artificial irradiation (Fig. 10a). Figure 12a–c shows that this signal does not saturate even at high microwave power as observed in [10].

### ESR-Based Age of Latest Event of The Ushikubi Fault from Calcareous Gouge

**Fig. 13:**
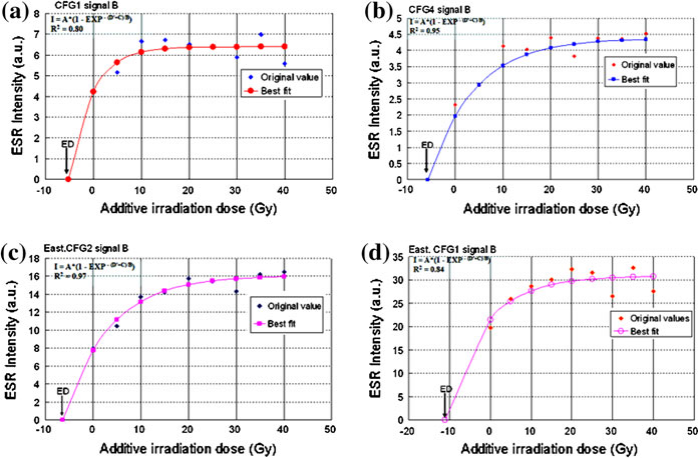
**a** Growth line and equivalent dose obtained from signal B in sample CFG1 after artificial irradiation. This signal gives a high correlation coefficient when compared with the others. **b** Growth line and equivalent dose obtained from signal B in sample CFG4 after artificial irradiation. The correlation coefficient in this sample is much more higher than in the other samples. **c** Growth line and equivalent dose obtained from signal B in sample East.CFG1. **d** Growth line and equivalent dose obtained from signal B in sample East.CFG2. The correlation coefficient is higher when compared with sample CFG1. The equivalent dose was obtained by extrapolation to the zero ordinate with the least-square fitting method. A, B and C, fitting parameters; *D*′, artificial irradiation; *R* ^2^, correlation coefficient

To estimate the ESR-based age (*T*_ESR_) of the latest event of the Ushikubi fault using calcareous fault gouge, the equivalent dose (ED) and the annual dose rate (*D*) were key parameters used as stated in [10]. The equivalent dose (Fig. 13a–d) was obtained by extrapolation of data points to the zero ordinate using the least-square fitting method. Although some of the signals showed saturation behavior with increasing dose, the data points were fitted into a simple saturation curve of Eq. (3).

3 $$ I = A\; \times \;\left( {1 - {\text{EXP}}^{{ - \, \left( {D' + C} \right)/B}} } \right), $$

where *A*, *B* and *C* are fitting parameters used in the calculation and *D*′ is the artificial irradiation dose (Gy). Moreover, it is possible to estimate the ED at low added dose since the signals did not show saturation behavior at that level. But to better understand the nature of the growth curve, fitting all the data points into a simple saturation growth curve is necessary. However, determining the ED with only three data points will not be enough to come out with a concrete conclusion.

**Fig. 14:**
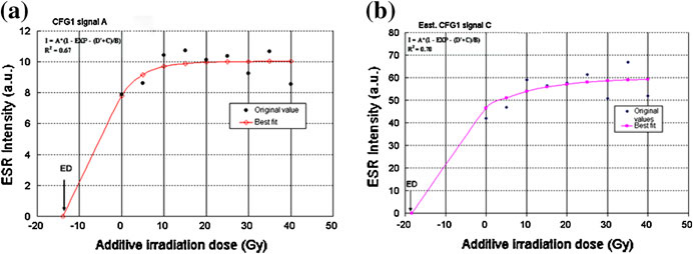
**a** Growth line and equivalent dose obtained from signal A in sample CFG1. The correlation coefficient is small as compared to that of signal B. **b** Growth line and equivalent dose obtained from signal C in sample East.CFG1. The correlation coefficient of this signal is also small

Since the degree of saturation varied between signals in different samples, signal B in the different samples was considered for the estimation of the equivalent dose, which was then used to determine the age of the latest event of the Ushikubi fault. This is because signal B had the highest correlation (*R* ^2^) coefficient and best fitting when compared with the signals A and C (Fig. 14a, b) in the samples investigated. More so, Ikeya et al. [10] stated that this signal is less visible in old samples. The annual dose values that were used in this study were adopted from the data of [6] and are given in Table 1, which shows that the annual dose rate values that range from 2.49 to 3.82 Gy/ka were calculated based on the concentration of ^238^U, ^232^Th and K_2_O. The uncertainty in the annual dose estimation arises from the point that the annual dose rates were estimated about the matrices of fault gouge, assuming radioactive equilibria and possible loss of radon and water content of 0–100 % and 0–10 %, respectively.

**Table 1:** Equivalent doses (ED) and ESR ages obtained from calcareous gouge of the Ushikubi fault

Sample code	Equivalent dose (ED) (Gy)	Annual dose (*D*) (Gy/ka)		Annual dose (*D*) (Gy/ka)	
		ESR ages (ka) (0 % water)		ESR ages (ka) (10 % water)	
		Signal B			
	Signal B	Radon loss 0 %	Radon loss 100 %	Radon loss 0 %	Radon loss 100 %
		(3.82)	(2.80)	(3.39)	(2.49)
CFG4	5.73	1.50	2.05	1.69	2.30
East.CFG2	6.4	1.68	2.29	1.89	2.57
East.CFG1	11.1	2.91	3.96	3.27	4.46
CFG1	5.2	1.36	1.86	1.53	2.09

Annual dose (*D*) (Gy/ka) values in bracket were adopted from the data of [6] from the Atotsugawa fault

*CFG* calcareous fault gouge from the central part of the Ushikubi fault, *East.CFG* calcareous fault gouge from the eastern part of the Ushikubi fault

The ESR ages for the calcareous gouges were then determined using Eq. (4).

4 $$ T_{\text{ESR}} = {\text{ED}}/D, $$

where ED is the equivalent dose obtained by the least-square method and *D* is the annual dose.

The ages obtained from calcareous gouge from the central part of the Ushikubi fault ranged from 1.4 to 1.5 ka, assuming no water and radon loss (Table 1). These results are consistent with the ages (700–1000 years) obtained indirectly from radiocarbon dating from trench excavation surveys by [15] indicating the effectiveness of the new method employed. Considering no water and radon loss, the ages obtained ranged from 1.7 to 2.9 ka in the eastern part of the Ushikubi fault (Table 1). According to indirect measurement by radiocarbon dating [17], the latest event along the northeastern part of Ushikubi fault (Ozorei) occurred about 500–700 years.

By taking the average of the ages obtained from both the central and the eastern part of the Ushikubi fault (1.4–1.5 ka and 1.7–2.9 ka, respectively), it was observed that the latest event on the fault occurred about 1.9 ka, which is consistent with the age of 1 ka obtained by the indirect method of radiocarbon measurement from trench excavation surveys. The observed agreement in the ESR-based calcareous gouge age in this study with the radiocarbon dating suggests that the novel method of using calcareous gouge ESR signal to determine latest event of active faults, which is the focus of this study, is probably a reliable tool for dating active fault events.

However, the age obtained in this study is slightly greater than the age obtained through the indirect radiocarbon dating method [15–17]. A relatively younger age value could be obtained if a smaller irradiation dose rate is used. Although the additive method is a standard method in the field of ESR, a high artificial irradiation dose rate (e.g., 50 Gy/h) can create fewer electron centers [27]. Ikeya et al. [10] stated that high dose rate effect leads to an erroneously high equivalent dose. This observation could be a contributing factor to the relatively older age value obtained in this study.

## Conclusion

ESR analysis showed that *g* factors (2.0060, 2.0033, and 2.0007) from surface gouge samples are different from *g* factors (2.0003, 2.0022, and 2.0066) from the core samples along the Ushikubi fault. Despite the depth-dependent difference in *g* factors, both the shallow and deep fault gouges showed signals that are indicative of A (${\text{SO}}_{2}^{\cdot{ - }}$), B (${\text{SO}}_{3}^{\cdot{ - }}$), and C (${\text{CO}}_{2}^{\cdot{ - }}$). Isochronal annealing experiment revealed a unique behavior in the ESR spectra of signal C in both the surface and the core samples in that a decrease in the signal intensity occurred at two stages indicating the presence of another signal (the $E_{1}^{\prime }$ defect centers of quartz). When the samples were irradiated artificially, some of the signals got saturated with increasing dose rate. Although the degree of saturation varied within signals in different samples, signal B was considered for the estimation of equivalent dose which was then used to determine the age of the latest event of the Ushikubi fault. For the first time, ESR method using signal B has been used to estimate the age of the Ushikubi fault from calcareous gouge. The mean age (1.9 ka) obtained is compatible with the indirect radiocarbon measurement method from trench excavation surveys along the fault. This proved the effectiveness of the method using calcareous gouge as a dating technique that measured the latest age of the fault activity directly. Although the age obtained in this study is slightly greater than the age obtained indirectly from radiocarbon dating, a maximum resolution of the latest fault event by this promising method could be obtained if pure carbonate gouge samples and a smaller irradiation dose rate can be used.

## References

[1] Suh EC, Ayonghe SN, Njumbe ES (2001). Episodes.

[2] Keller EA, Pinter N (2002). Active Tectonics, Earthquake, Uplift and Landscape.

[3] Suganuma K (2006). Q. Rev..

[4] Yamasaki E (2012). Momentum.

[5] Ikeya M, Miki T, Tanaka K (1982). Science.

[6] Fukuchi T, Mie H, Okubo S, Imai N (2002). Adv. ESR Appl..

[7] Toyoda S, Schwarcz HP (1997). Radiat. Meas..

[8] Hataya R, Tanaka K, Miki T (1997). Quat. Sci. Rev. (Quat. Geochronol.).

[9] J. S. Noller, J. M. Sowers, W. R. Lettis, in *Quaternary Geochronology: Methods and Applications*, (AGU Reference Shelf 2000), 4, 581 doi:10.1029/RF004

[10] Ikeya M, Zimmerman MR, Whitehead N (1993). New Applications of Electron Spin Resonance: Dating, Dosimetry and Microscopy.

[11] I.S. Stewart, P.L. Hancock, *Neotectonics in Continental Deformation*, ed. by P.L. Hancock, (Oxford Pergamon Press, London, 1994), pp. 341–399

[12] Fu G, Zheng B, Yoshio T, Miyahara M, Kuno A, Matsuo M, Miyashita Y (2008). Hyperfine Interact.

[13] R. Doke, A. Takeuchi, in *Geodynamics of Atotsugawa Fault System*, ed. by M. Ando (TERRAPUB, Tokyo, 2007) pp. 11–16

[14] A. Takeuchi, A. Takebe, H. Ongirad, R. Doke, in *Geodynamics of Atotsugawa Fault System*, ed. by M. Ando (TERRAPUB Tokyo, 2007) pp. 1–10

[15] Y. Miyashita, T. Yoshioka, T. Kuwabara, M. Saitoh, K. Kobayashi, Y. Kariya, K. Fujita, T. Chiba, in *Annual Report on Active Fault and Paleoearthquake Research*, 4, 131–142 (2004), (in Japanese with English abstract)

[16] Y. Miyashita, T. Yoshioka, M. Nikaido, N. Takase, T. Tachibana, in *Annual Report on Active Fault and Paleoearthquake Research*, 4, 131–142 (2004), (in Japanese with English abstract)

[17] Y. Miyashita, K. Kobayashi, M. Nikaido, N. Takase, T. Ojiri, in *Annual Report on Active Fault and Paleoearthquake Research*, **5**, 85–93 (2005), (in Japanese with English abstract)

[18] A.M. Rossi, G. Poupearu, Appl. Radiat. Isot. 40, 1133–1137 (1989)

[19] Molodkov A (1988). Quat. Sci. Rev..

[20] Barabas M, Bach A, Mudelsee M, Mangini A (1992). Quat. Sci. Rev..

[21] Miki T, Kai A (1991). Jpn. J. Appl. Phys..

[22] Engin B, Guven O, Koksal F (1999). Appl. Radiat. Isot..

[23] Piroulle F, Bahain JJ, Falgueres C, Dolo JM (2007). Quat. Geochronol..

[24] Angiolillo PJ, Graneto N (2008). Radiat. Phys. Chem..

[25] Brumy S, Yoshida H (1995). Radiat. Meas..

[26] Grun R, De Canniere P (1984). J. Radioanal. Nucl. Chem..

[27] P.J. Groom, S.A. Durrani, K.A.R. Kazal, S.W.S. Mckeever, PACT J, 2, 200–210 (1978)

[28] N. Yamada, S. Harayama, F. Takizawa, T. Kato, T. Hiroshima, M. Komazawa, Geological map of Japan, 1:200,000, Takayama. Kanazawa (Geological Survey of Japan, 1988)

[29] K. Kano, S. Harayama, M. Yamamoto, M. Takeuchi, K. Uto, M. Komazawa, T. Hiroshima, S. Sudo, Geological map of Japan 1:200,000, Kanazawa (Geological Survey of Japan, 1999) (in Japanese)

[30] Suzuki K, Toda S, Kusunoki K, Fujimitsu Y, Mogi T, Jomori A (2000). J. Eng. Geol..

